# Increased Salivary IL-1 Beta Level Is Associated with Poor Sleep Quality in University Students

**DOI:** 10.3390/diseases11040136

**Published:** 2023-10-05

**Authors:** María Luisa Ballestar-Tarín, Vanessa Ibáñez-del Valle, Mayra Alejandra Mafla-España, Omar Cauli, Rut Navarro-Martínez

**Affiliations:** 1 Department of Nursing, University of Valencia, 46010 Valencia, Spain; m.luisa.ballestar@uv.es (M.L.B.-T.); maria.v.ibanez@uv.es (V.I.-d.V.); maymaes@alumni.uv.es (M.A.M.-E.); rut.navarro@uv.es (R.N.-M.); 2 Nursing Care and Education Research Group (GRIECE), GIUV2019-456, Nursing Department, Universitat de Valencia, 46010 Valencia, Spain; 3 Frailty Research Organized Group (FROG), University of Valencia, 46010 Valencia, Spain; 4 Department of Hematology, University General Hospital, 46014 Valencia, Spain

**Keywords:** students, sleep, quality, salivary biomarkers

## Abstract

Poor sleep quality is a major public health concern for all ages. In particular, university students often face stress levels and changes in social life habits that negatively influence their quality of sleep. This could be associated with psychological well-being in terms of anxiety and depressive symptoms, stress levels, and a poor self-perceived health status. The increases in the pro-inflammatory cytokine interleukin 6 (IL-6), IL-1 beta (IL-1β), and tumor necrosis factor alpha (TNFα), in blood have been linked to poor sleep quality in many diseases, but data on salivary cytokine levels in students are missing or are seldom analyzed. In this study we determined the quality of sleep in a sample of university students and the role of psychological assessment and factors affecting sleep (alcohol intake, tobacco, consumption of stimulant drinks, exercise, and body mass index). We also aimed to shed new light on the associations between sleep quality and salivary inflammatory cytokines (IL-1β, IL-6, and TNFα). Sleep quality was measured with the Athens Insomnia Scale (AIS) and Pittsburgh Sleep Quality Index (PSQI). Perceived stress was assessed using Cohen’s Perceived Stress Scale (PSS), and the Goldberg Anxiety and Depression Scale (GADS) was used to assess the level of anxiety or depression. Perceived health status was measured with a visual analogue. Saliva samples was taken in the morning and the inflammatory cytokines was measured via enzyme-linked immunoassay. There was a direct and significant association between the salivary IL-1β concentration and AIS score (r = 0.248; *p* = 0.038, Pearson correlation) and Pittsburgh scale score (r = 0.274; *p* = 0.022, Pearson correlation). The relationship between IL-1β and AIS controlling for sex, age, and chronic disease, is still significant (r = 0.260; *p* = 0.033). The relationship between IL-1β and PSQI controlling for the influence of these variables is also significant (r = 0.279; *p* = 0.022). Salivary IL-1β concentrations were not significantly associated with any of the scores of the other psychological assessments (PSS, anxiety, depression symptoms, or self-perceived health). Salivary TNFα was significantly and inversely associated with self-perceived health (r = −0.259; *p* = 0.033, Pearson correlation), but the salivary IL-6 concentration was not associated with any of the sleep quality scale or psychological assessment scores. Our results provide a novel relationship between pro-inflammatory cytokine IL-1β in saliva and poor sleep quality. However, the role of inflammation in poor sleep quality requires further study to identify strategies that could lower inflammation and thus, likely improve sleep quality.

## 1. Introduction

Sleep is essential for maintaining adequate physical and mental health. Despite this, as reported in a meta-analysis conducted in the United States, only one third of individuals with insomnia consult about this problem, and only 5% seek treatment [1]. The predominant complaint is difficulty initiating or maintaining sleep or nonrestorative sleep [2]. This difficulty in sleeping generates excessive worry in the sufferer, contributing to the perpetuation of the problem [3]. A review on the epidemiology of insomnia published in 2002 reported that the prevalence of insomnia varies between 5% and 50% depending on the diagnostic criteria used [4]. Similar to the general population, insomnia in the university population represents a frequent problem which can affect well-being and academic performance. One of the main contributors to insomnia in university students is academic stress. Many students feel pressure to perform well academically, which can lead to anxiety and difficulty sleeping. Additionally, the workload and demands of university life can leave students feeling overwhelmed and stressed, which can further exacerbate sleep problems.

Social pressures can also play a role in insomnia among university students. Indeed, many students are living away from home for the first time and may feel pressure to socialize and participate in extracurricular activities. This can lead to irregular sleep schedules and a lack of routine, which can make it difficult to establish healthy sleep habits. Changes in sleep patterns can also contribute to insomnia in university students. Many students may stay up late studying or socializing, leading to a delay in their natural sleep–wake cycle [5]. Poor sleep hygiene is often responsible for insomnia problems in university students [6], characterized by poorer sleep quality, longer sleep latency, more sleep disturbances, and greater daytime dysfunction, especially among caffeine, alcohol, and tobacco users [7]. In addition, it has been shown that the absence of adequate sleep may affect academic performance [8] and has been associated with an increased likelihood of complications such as obesity [9], anxiety, stress, depression, and increased consumption of hypnotics and stimulants [10]. Furthermore, nursing students may be particularly vulnerable to sleep disorders because in their future professional lives, many of them will work night shifts which can maintain or worsen their poor sleep quality.

Quality of sleep can be affected by several bio-psycho-social factors. Among the biological factors, inflammation and inflammatory molecules have been associated with poor sleep quality and sleep disorders. Inflammation is a natural response of the immune system to infection, injury, or stressful stimulus. During sleep, the body releases cytokines, proteins that help the immune system to fight off infections, inflammation, and stress. There is a solid body of evidence [11,12,13] that demonstrates that sleep deprivation can lead to an increase in peripheral inflammation in the body, which can contribute to the development or worsening of several chronic diseases. Indeed, studies have shown that people who get less sleep or poor-quality sleep are more likely to have higher levels of inflammation markers in their bloodstream. Conversely, reducing inflammation in the body through lifestyle changes such as diet, exercise, and stress management can improve sleep quality.

Salivary biomarkers are biological molecules that can be measured in saliva and used as indicators of various physiological and pathological conditions. In the context of university students, salivary biomarkers can be used to assess their overall health status, stress levels, and academic performance. Overall, salivary biomarkers can provide valuable insights into the health and well-being of university students and may help identify individuals who are at risk of poor health outcomes or academic performance. Of note, an increase in the concentration of inflammatory markers such as interleukin 6 (IL-6), IL-1 beta (IL-1β), and tumor necrosis factor alpha (TNFα), in blood samples has been associated with poor sleep quality [14,15]. Furthermore, in young adults, a good night’s sleep is associated with decreased daytime secretion of IL-6 [16,17,18]. Sleep deprivation increases daytime IL-6 and causes somnolence and fatigue during the next day, whereas in the subsequent days, there is a decrease in nighttime IL-6 production which is associated with deeper sleep [17]. Morning IL-6 levels in blood are positively correlated with REM latency after sleep onset and negatively correlated with sleep efficiency and slow wave sleep [16]. Finally, blood TNFα levels decrease during sleep in healthy young men [19]. There is evidence in many populations that the concentration of inflammatory cytokines in saliva is significantly associated with their concentration in blood stream in most studies [20,21,22].

The hypothesis of the study was that levels of salivary inflammatory cytokines (IL-1β, IL-6 and TNFα) are related to sleep quality in university students. The aims of this study were: (1) to analyze insomnia in a population of university students and the association between sleep quality and several factors such as depressive symptoms and self-perceived stress, drinking beverages containing caffeine, smoking, and alcohol intake; (2) to evaluate whether the concentration of salivary inflammatory markers IL-1β, IL-6, and TNFα is associated with insomnia and the discrimination accuracy of these cytokines to classify individuals with or without insomnia.

## 2. Materials and Methods

### 2.1. Study Design and Population

This research study had a cross-sectional design and was conducted between September 2021 and June 2022 with nursing degree students at the University of Valencia (Spain). The research complied with the requirements of the Declaration of Helsinki, and the entire study protocol was approved by the University of Valencia ethics committee (protocol number 1232539, released on 21 May 2020).

### 2.2. Study Sample

The study population or target population comprised students in the first year of the nursing degree at the University of Valencia. The sample was obtained through non-probabilistic convenience sampling whereby the participants were selected according to their accessibility. The sample size was calculated using the population estimation, considering that 240 students were in the first year of the course. Thus, based on a previous study [23], a sample size of 68 randomly selected participants was sufficient to estimate a population percentage of around 40% with 95% confidence and a precision of ±10%. Sample size estimation was also calculated using a correlation coefficient: accepting an alpha risk of 0.05 and a beta risk of 0.2 in a two-sided test, the result is 59, considering an acceptable correlation coefficient between the score of the psychometric questionnaires and the concentration of inflammatory cytokines in saliva of 0.4. A drop-out rate of 20% was anticipated [24]. The final sample of the research consisted of 72 participants. Before conducting the study assessments and collecting the saliva sample, we asked the students if they had caries, toothache, or gum problems and, if so, no saliva sample was collected.

### 2.3. Information Collection Procedure

The students completed an anonymous questionnaire that included socio-demographic variables (age, sex, and marital, cohabitation, and employment status) as well as lifestyle data (engagement in exercise; consumption of stimulant drinks such as coffee, tea, or cola; consumption of tobacco or alcohol) and health data (chronic diseases and perceived health status), the Athens Insomnia Scale (AIS), Pittsburgh Sleep Quality Index (PSQI), Cohen Perceived Stress Scale (PSS), and Goldberg Anxiety and Depression Scale (GADS). The students were asked whether they suffered from any chronic illnesses. If so, they had to specify which illness they suffered from. The following diseases were included in the list: anxiety, depression, diabetes, cardiovascular disease, gastrointestinal disease, chronic respiratory disease, and others (in this case, the students had to indicate which disease it was). Alcohol consumption was assessed using the AUDIT-C3. In addition, a saliva sample was also collected to measure lL-1β, IL-6, and TNFα levels.

### 2.4. Evaluation of Sleep Quality

The AIS was developed by Soldatos et al. (2000) to assess insomnia according to the ICD-10 criteria [25]. It consists of 8 Likert-type response items ranging from 0 (no problem) to 3 (a serious problem), and the overall scale score ranges from 0 to 24 points. Respondents answer according to whether they have experienced the difficulties mentioned at least three times a week in the month prior. The cut-off point is set at 6 points and so poor sleep quality is considered for scores exceeding 6 points and normal sleep for scores under 6 points.

The PSQI is a self-administered questionnaire containing a total of 19 items that provides an overall rating of sleep quality. These 19 items combine to form 7 areas with corresponding scores (sleep quality, sleep latency, sleep duration, habitual sleep efficiency, sleep disturbance, use of sleeping medication, and daytime dysfunction), each with a range of 0 to 3 points. A score of 0 indicates ease, while a score of 3 indicates severe difficulty for each area. The scores in the 7 areas were summed to give an overall score which ranged from 0 to 21 points where 0 indicates ease of sleeping, and 21 indicates severe difficulty in all areas. A score of 5 was originally used to categorize good (≤5) and bad sleepers (>5).

### 2.5. Evaluation of Stress, Depression and Anxiety Symptoms, and Self-Perceived Health

Perceived stress was assessed with Cohen’s PSS scale, which has been validated in Spanish. The PSS is the best-known instrument for measuring perceived psychological stress, i.e., the degree to which everyday life situations are rated as stressful. It is a scale consisting of 10 items that assess how often the respondent has felt a certain way in the previous month. Responses are scored as 0 (never), 1 (hardly ever), 2 (sometimes), 3 (quite a lot), or 4 (very often). However, items 4, 5, 7, and 8 are scored inversely or inverted. The total score ranges from 0 to 40 points, with higher scores indicating a higher degree of perceived stress. Some authors consider a score of 14 as the cut-off point [26,27].

The GADS, which has been validated in Spanish, was used to assess the level of anxiety or depression. It orients the diagnosis towards anxiety or depression (or both in mixed cases) and discriminates between them and quantifies their respective intensities. This scale consists of two subscales, one for anxiety and one for depression. Each of the subscales comprises 9 dichotomous response items (YES or NO) to determine whether the respondent has experienced any of the mentioned symptoms in the 2 weeks prior. The cut-off points are 4 or more items or affirmative responses for the anxiety scale and 2 or more for the depression scale. The higher the score, the more severe the problem (the maximum possible score is 9 points for each of the subscales). Given that no cut-off point has been established for the total score, we used a value of 11, which is equivalent to the third quartile.

Perceived health status has been found to be a good predictor of actual health status [28] and refers to the individual self-assessment of each participant’s health status. In this study, it was assessed with a visual analogue scale from 1 to 5 points, with a higher score corresponding to better health status. Self-perceived health (SPH) is included in one of the 6 groups of determinants of the active ageing model proposed by the World Health Organization [29].

### 2.6. Other Factors Influencing Sleep Quality

We evaluated the frequency at which participants engaged in exercise (number of days practicing any sport each week) and consumption of stimulant drinks, tobacco, and alcohol. For alcohol intake, we used the AUDIT questionnaire as a simple screening method to identify heavy alcohol consumption, as recommended by the World Health Organization. The AUDIT-C3 comprises 3 questions that are scored from 0 to 4. The cut-off point for males is set at 7 points and for females at 5 points [30].

### 2.7. Saliva Collection and Cytokines Analysis

Participants were asked to refrain from smoking, eating, drinking, or undergoing oral hygiene procedures for at least one hour prior to sample collection. Saliva samples were obtained in the morning between 10:00 and 12 p.m. using the synthetic Salivette^®^ system (Sarstedt, Germany). Each sample was centrifuged to remove mucins, insoluble material, and cellular debris, and the supernatant was aliquoted into Eppendorf tubes and frozen (−80 °C) until further analysis. The samples (100 μL) were brought to room temperature, and ELISA assays were performed in duplicates using the commercial Abcam High Sensitivity Human Elisa Kit for IL-6 (ab46042, Cambridge, UK), IL-1β (Ab214025, Cambridge, UK), and TNFα (ab181421, Cambridge, UK) according to the manufacturer’s instructions (Abcam, Amsterdam, The Netherlands). Changes in color intensity and absorbance at 450 nm and 490 nm were read using an ELISA microplate reader, and a standard curve was prepared by plotting absorbance readings of standards against their concentrations.

### 2.8. Statistical Analysis

Quantitative variables were described using descriptive statistics, including measures of central tendency (mean), standard error of the mean (SEM), and range values. The normal distribution of each variable was estimated with the Kolmogorov–Smirnov test. Student *t*-tests and ANOVA were used to compare means between quantitative variables, and chi-square tests were used to compare proportions. The relationship between quantitative variables was tested with Pearson’s correlation coefficients. Statistical significance was set at *p* < 0.05. All the statistical analyses were performed using SPSS software (version 28.0, IBM Corp., Armonk, NY, USA).

## 3. Results

### 3.1. Socio-Demographic Characteristics of the Study Sample

The sample comprised 72 students from the nursing degree at the University of Valencia; 79.2% were female (*n* = 57) and 20.8% were male (*n* = 15). The mean age of the participants was 20 (SD = 5.569). Seventy-five percent of the students were single, and they mainly lived with their families (70.8%, *n* = 51); 86.3% of the sample were full-time students (not working) (*n* = 62), and 13.9% were in part-time or full-time employment while studying (*n* = 10). There were no differences between men and women (χ^2^ = 1.343, *p* = 0.247), employment status (χ^2^ = 2.302, *p* = 0.316), or age (t = −0.421, *p* = 0.338), but there was a significant difference between cohabitation types (family versus other categories: χ^2^ = 8.019, *p* = 0.046).

### 3.2. Sleep Quality

The AIS items assess different aspects of sleep. As shown in Table 1, sleep induction, sleep duration, daytime sleepiness, and sleep duration had the worst scores; 22.2% (*n* = 16) of the participants indicated that they had a moderate problem with sleep induction. The AIS items most affected were that 20.8% (*n* = 15) of the students indicated a markedly insufficient overall sleep duration and 16.7% (*n* = 12) described considerable daytime sleepiness.

Measurement of sleep quality with the AIS showed a mean score of 5.43 (SD = 3.29). According to the AIS cut-off of 6 points, 44.4% of the participants reported insomnia (*n* = 32), while 55.6% reported normal sleep (*n* = 40). The ‘difficulty in sleeping’ dimension had the lowest mean score among the participants. No differences were observed between men and women (χ^2^ = 0.152, *p* = 0.697), cohabitation types (family versus other categories: χ^2^ = 1.926, *p* = 0.588), working or not (χ^2^ = 1.327, *p* = 0.515), or ages (t = −1.294, *p* = 0.101).

Measurement of sleep quality with the PSQI showed a mean score of 6.75 (SD = 3.236). Considering the cut-off of 5 points, 59.7% (*n* = 43) of the participants had poor sleep quality compared to the 40.3% (*n* = 29) who slept well. There were no differences between men and women (χ^2^ = 1.343, *p* = 0.247). The PSQI components with the worst scores were daytime dysfunction, sleep latency, and sleep duration, which 36.1–41.7% of the individuals considered to be quite or very bad (Table 2).

### 3.3. Stress, Anxiety and Depression Symptoms, Self-Perceived Health, and Their Association with Sleep Quality

The mean score obtained with the PSS was 17.30 (SD = 6.312). No differences were observed between men and women (t = −1.135, *p* = 0.129). Considering a score of 21 (third quartile) as the cut-off, 37.5% (*n* = 27) of the participants reported stress symptoms, with no differences between men and women (χ^2^ = 2.476, *p* = 0.116). However, if we instead consider 14 as the cut-off, 73.6% (*n* = 53) reported stress symptoms. Regarding the anxiety–depression symptoms, a cut-off of 4 points or more was used for the anxiety subscale and 2 or more for the depression subscale. The higher the scores, the more severe the problem (with the maximum possible being 9 in each of the subscales) and the maximum for both scales being 18. The overall mean score of the scale was 7.972 (SD = 4.80). For the anxiety subscale, the mean was 4.819 (SD = 3.031) and for the depression subscale, the mean was 3.153 (SD = 2.504); 34.7% (*n* = 25) obtained a score equal to or higher than 11 on the PSS.

Considering a cut-off of 4 points or more on the anxiety subscale, 54.9% (*n* = 40) of the participants were at risk of suffering from anxiety. In the case of depression, if we considered the cut-off as 2 or more points, 43.4% (*n* = 31) were at risk of suffering from depression; 33.3% (*n* = 24) of the sample showed symptoms of anxiety and depression, with the perceived health status of 3.94 (SD = 0.611; range = 2–5). Based on the suggested cut-off of >3 points, 85.7% (*n* = 62) of the participants stated they had good self-perceived health. Finally, quality of sleep scores on both the AIS and PSQI were significantly correlated with the psychological assessment scores (stress levels, anxiety and depression symptoms, and self-perceived health status; Table 3).

### 3.4. Factors Affecting Sleep and Their Association with Sleep Quality

A total of 69.4% (*n* = 50) of the students engaged in sports for a mean of 2.68 days per week (SD = 2.18). Engaging in exercise was significantly more common in men than in women (χ^2^ = 8.34, *p* < 0.004). In addition, 84.7% (*n* = 61) of the participants were non-smokers; only 12.5% (*n* = 12) said they did not drink alcohol whereas, according to the AUDIT-C questionnaire, 56.9% (*n* = 41) of the participants showed a risky drinking pattern; 72.2% (*n* = 52) of the students consumed stimulant beverages. A total of 81.4% were a normal weight, 15.7% (*n* = 11) were overweight, and 2.9% (*n* = 2) were underweight. Moreover, 15.5% (*n* = 11) of the sample stated they suffered from a chronic illness (four students had allergies, two had hypothyroidism, two had scoliosis, one had anemia, and two had celiac disease).

No significant differences were observed in the AIS score between people with or without a chronic disease (t = −1.140, *p* = 0.129). Nor was the AIS significantly different between participants who were a normal weight or were overweight (t = −0.865, *p* = 0.195), those who consumed stimulant drinks or not (t = −0.424, *p* = 0.673), smokers and non-smokers (t = −0.140, *p* = 0.889), or who consumed alcohol (t = −0.354, *p* = 0.705). However, there were differences in the AIS scores between those who did or did not participate in sports (t = 2.311, *p* = 0.002), with the former obtaining lower AIS scores (slept better) than the latter.

No differences were observed in the score obtained for the PSQI between people with or without a chronic disease (t = −1.090, *p* = 0.280), who were a normal weight compared to those who were overweight (t = −0.315, *p* = 0.754), people who engaged in exercise or not (t = 1.475, *p* = 0.145), consumed stimulant beverages or not (t = −0.485, *p* = 0.625), smoked or not (t = −0.277, *p* = 0.783), or who drank alcohol or not (t = −191, *p* = 0.849).

### 3.5. Salivary Cytokines and Their Association with Sleep Quality

The mean concentration of IL-1β, IL-6, and TNFα were 257.20 pg/mL (SD = 195.60), 30.37 pg/dL (SD = 33.25), and 31.88 pg/dL (SD = 37.15), respectively. There is a significant correlation between IL-1β and IL-6 (r = 0.362; *p* = 0.002) but no significant correlations between TNFα and IL-1β (r = −0.194; *p* = 0.109) or TNFα and IL-6 (r = −0.084; *p* = 0.494).

As shown in Table 4, Pearson correlations showed direct significant associations between the salivary IL-1β concentration and AIS (r = 0.248; *p* = 0.038) and PSQI scores (r = 0.274; *p* = 0.022). However, the salivary IL-1β concentration was not significantly associated with any of the other psychological assessments (self-perceived stress level, anxiety, depression symptoms, or self-perceived health). In addition, salivary TNFα was significantly and inversely associated with self-perceived health (r = −0.259; *p* = 0.033). The salivary IL-6 concentration was not associated with any of the sleep quality or psychological assessment scale scores (Table 4).

**Figure 1:**
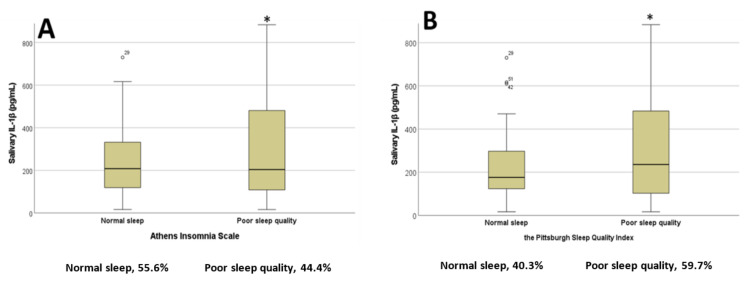
Salivary interleukin 1 beta concentrations in students with normal or poor sleep quality based on the (**A**) Athens Insomnia Scale and (**B**) Pittsburgh Sleep Quality Index; * *p* < 0.05. Percentages of participants in each category are represented below the x axis (*n* = 72). Abbreviations: interleukin 1 beta, lL-1β.

As shown in Figure 1A,B, when using the proposed cut-off scores for the AIS and PSQI scales we found that students with poor sleep quality had significantly higher salivary IL-1β concentrations compared to those with normal sleep scores (t = 1.692, *p* = 0.04 for AIS; t = −1.760, *p* = 0.041 for PSQI).

**Figure 2:**
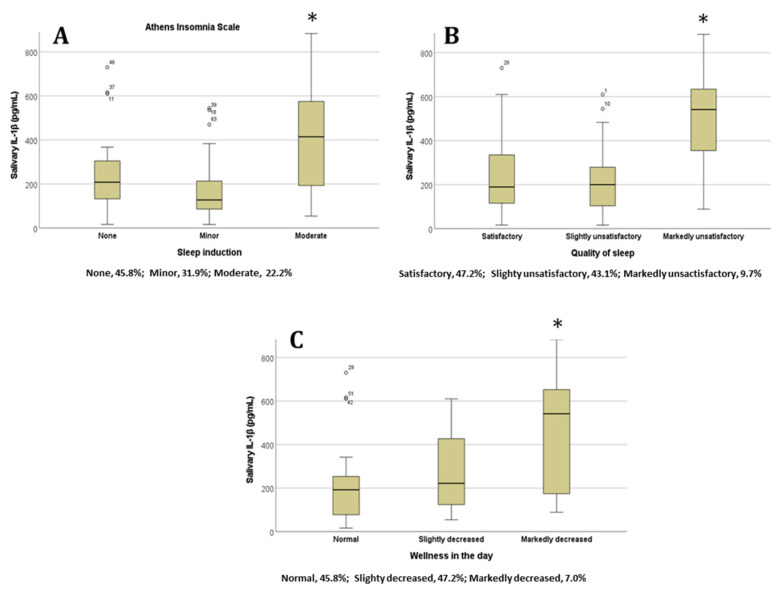
Salivary IL-1β concentrations in students based on their answers to the Athens Insomnia Scale items (**A**) ‘Sleep induction’, (**B**) ‘Overall quality of sleep’, and (**C**) ‘Sense of well-being during the day’; * *p* < 0.05. Percentages of participants in each category are represented below the x axis (n = 72). Abbreviations: interleukin 1 beta, lL-1β.

In order to analyze the sleep quality items, which were mostly related to different IL-1β concentrations, we examined each item of the AIS and PSQI for significant differences across the categories. For the AIS, the IL-1β concentration was significantly higher for the ‘Sleep induction’ (Figure 2A), ‘Overall quality of sleep’ (Figure 2B), and ‘Sense of well-being during the day’ (Figure 2C) item categories when the students had selected the ‘Moderate problem’ option (*p* < 0.05 in all cases, ANOVA test).

**Figure 3:**
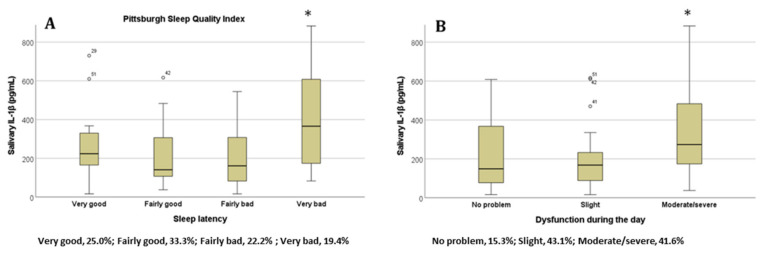
The salivary IL-1β concentration in students based on their answers to the items (**A**) ‘Sleep latency’ and (**B**) ‘Daytime dysfunction’ on the Pittsburgh Sleep Quality Index; * *p* < 0.05. Percentages of participants in each category are represented below the x axis (*n* = 72). Abbreviations: interleukin 1 beta, lL-1β.

For the PSQI, we also found significantly higher IL-1β concentrations for the ‘Sleep latency’ (Figure 3A) and ‘Daytime dysfunction’ (Figure 3B) items when students selected the ‘very bad’ and ‘moderate/severe’ options for each of these items, respectively (*p* < 0.05 in all cases, ANOVA test). The results show that the partial correlations between IL-1beta and sleep quality (AIS and PSQI) are still significant when taking into account possible confounders. The relationship between IL-1β and Athens Insomnia Scale controlling for sex, age, and chronic disease, is still significant (r = 0.260; *p* = 0.033). The relationship between IL-1β and Pittsburgh Sleep Quality Index (PSQI) controlling for the influence of these variables is also significant (r = 0.279; *p* = 0.022). Sleep quality was not significantly associated with salivary Il-6 or TNF-alpha concentration (see supplementary Figure S1).

## 4. Discussion

As found in previous work [23], we found that poor sleep quality was a highly prevalent problem among university students. To the best of our knowledge, this present study is the first to explore and identify associations between the level of IL-1β measured in saliva and sleep quality in this population. In addition, we have observed that environmental, lifestyle, and mood factors are involved in the regulation of sleep in university students, which we have previously reported to have a poor sleep quality [23]. The prevalence rates of insomnia symptoms in our study population reached 44.4% and 59.7% for the AIS and PSQI, respectively, and were considerably higher than the rates reported elsewhere for the age-matched (20–29 years) general population [8,31,32]. Also, coinciding with previous findings [29,30], we found that the most frequent symptoms of insomnia were delayed sleep onset, insufficient sleep duration, and daytime sleepiness or dysfunction. Indeed, prolonged sleep latency can result in shorter sleep periods which can lead to daytime sleepiness and can negatively affect daily functioning [33].

University students are particularly susceptible to sleep difficulties because of their often-inconsistent sleep schedules derived from frequent late-night socializing, class schedules that vary throughout the week, and late-night studying [34]. In addition, the frequent use of mobile devices at bedtime, as well as the consumption of coffee or other stimulants, is very common among university students and can also negatively affect their sleep [35,36]. Similarly, uncomfortable sleep environments or engaging in activities other than sleeping in the bedroom (e.g., watching TV or a mobile device) can influence sleep quality [34]. The latter, as well as having less living space, could explain the worse quality of sleep observed among students who live in university residences or who share a room with other students. Similarly, due to the outbreak of SARS-CoV-2 (COVID-19) in 2019, the various outbreak prevention and control measures (for example, work stoppages, school closures, and lockdown management) applied have increased the prevalence of the symptoms of insomnia in this population [37].

In our analysis, university students with more symptoms of insomnia showed higher levels of IL-1β in saliva. IL-1β has been implicated in the physiological promotion of sleep and is related to other sleep-regulating substances [38]. In addition, like our findings, an increase in IL-1β has also been described in sleep-deprived animal models [39]. This may be a physiological defense mechanism to induce sleep and recover normal physiological function. Of note, IL-1β can stimulate the hypothalamic–pituitary–adrenocortical axis by activating the sympathetic nervous system (SNS) with the consequent production of cortisol. The latter generates an anti-inflammatory response which regulates IL-1β production when its levels are low [40]. While this inflammation control occurs under basal conditions, a sustained increase in IL-1β as a consequence of a persistent lack of sleep alters the body’s stress response system. In turn, this generates a state of cortisol resistance, whereby the increase in IL-1β transforms this inflammatory signal into nervous signals. The latter can induce increases in NF-κB, inflammatory gene expression, and the production of proinflammatory cytokines, including IL-1β, thus producing a positive feedback loop with greater increases in IL-1β levels [41,42].

Given that normal nocturnal sleep periods are characterized by a fall in SNS activity [43], activation of the sympathetic effector pathway is a biologically plausible mechanism to explain the associations between sleep disturbance and increased expression of inflammatory markers such as IL-1β [12], by promoting delayed sleep because of hyperarousal [20]. In fact, this could also explain the significantly higher concentration of IL-1β observed in students who indicated that their ‘Sleep latency’ was very bad or who said they had a moderate ‘Sleep induction’ problem on the PSQI and AIS scales, respectively. It should be emphasized that young university students generally present a circadian rhythm of delayed sleep [44] because they secrete melatonin later in the night than adults. This makes them naturally inclined to go to bed and to sleep later and so, in addition to an increased risk of insufficient sleep, they may also be more sensitive to the effects of IL-1β associated with a sustained lack of sleep. Zhai et al. (2022) showed that those students who presented an evening chronotype and preferred to sleep and get up late, not only slept for shorter periods but also presented higher serum levels of inflammatory markers such as IL-1β [18].

Our results add to the existing evidence that sleep deprivation increases the inflammatory response in healthy people. Because sleep and inflammation are bidirectionally linked, it is possible that various factors related to higher overall inflammation—associated with shorter sleep duration and later sleep times—could have influenced the poorer sleep quality in our participants. These include gut microbiota [45], high levels of adiposity [46], and lifestyle behaviors such as low fruit and vegetable consumption and reduced physical activity [47,48]. In fact, the present study found that students who engaged in some exercise had lower blood pressure according to the AIS (they slept better). Indeed, given that we observed lower levels of IL-1β in participants who performed physical exercise, this association between physical activity and sleep could be mediated by inflammation. In turn, these results also reinforce the link between sleep and inflammation by suggesting how, through the practice of regular and moderate exercise, markers of systemic inflammation are reduced and consequently, sleep quality is improved.

The above is especially important because physical exercise can be used as a non-pharmacological tool to optimize sleep quality in this population. In addition, the practice of physical exercise contributes to improving sleep quality by improving mental health [49]. In fact, we observed that participants in our work with affective dysfunction (anxiety/depression) tended to be less physically active and that students with greater symptoms of anxiety and depression showed poorer sleep quality. Furthermore, current evidence suggests that experiences of dreams often occur before the symptoms of depression and anxiety and may be a prodromal symptom of subsequent affective disorders [50]. In this sense, the prevention and reduction of sleep problems may be an appropriate and cost-effective way to reduce the rates of depression and anxiety in this population.

Likewise, here we observed an association between self-perceived stress and poor sleep in our student cohort. Perceived stress appears to be an important risk factor for poor sleep quality in this population group [51]. The university context includes stress-related events and situations such as leaving home, adjusting to new social situations, and dealing with high levels of academic pressure. This psychological stress, which is mediated through neuroendocrine alterations, causes an increase in awakenings as well as a delay in sleep that, in turn, is associated with hyperarousal [18]. However, once sleep problems are established, the daytime consequences of poor sleep interfere with the ability to cope with stressors, which contributes to increasing stress levels, making it even more difficult to fall asleep [51]. In this sense, interventions that improve sleep, especially during periods of high stress such as excessive academic pressure, can potentially improve student well-being and, consequently, benefit academic performance.

In this present study we also observed a positive association between sleep quality and self-perceived health status. It seems reasonable that dysfunction or daytime sleepiness related to a lack of sleep could have contributed to poorer self-reported health. This self-assessment is subjective, but considered a reliable indicator of the objective state of health of the population [52], thus existing poor health could have disturbed sleep in our participants. In this sense, we observed that the mood associated with poor sleep quality caused a negative bias in the health state evaluations of our students. Indeed, previous studies have shown that common psychological problems (e.g., depression and anxiety) are associated with self-rated health [53]. Thus, our findings suggest that affective disturbances, through poor sleep quality, may exert effects on self-perceived health. Hence, intervening to improve sleep quality in this population can reduce depression and anxiety symptoms, which in turn can increase the health status self-assessment outcomes.

Also of note, salivary TNFα was significantly and inversely associated with self-perceived health in our work. To the best of our knowledge, no studies have yet evaluated the correlation between self-perceived health and salivary TNFα levels. Nonetheless, the study by Lekanderet al. (2004) examined self-rated health and determined circulating levels of TNFα and other inflammatory biomarkers from plasma samples in primary healthcare patients [54]. They measured self-rated health using the following question: “How would you rate your general state of health?”. The response alternatives (coded 1 to 5) were very good, quite good, neither good nor poor, quite poor, and poor. The levels of inflammatory cytokines were higher in people with poorer subjective health, and there was a strongly significant association between self-rated health and TNFα levels. We did not find any other studies that directly measured the association between self-perceived health and inflammatory biomarkers. However, some work has studied the association between TNFα and other variables related to poorer subjective health such as pain, fatigue, or executive dysfunction.

In this sense, the article by Bautmans et al. (2008) [55] reported results from an explorative study investigating the relationships between muscle endurance and self-perceived fatigue, mobility, and circulating inflammatory parameters in elderly nursing home residents. The results of this work showed that higher salivary TNFα levels were related to worse fatigue resistance and poorer grip in the male residents [55]. Moreover, He et al. reported recently similar results when they evaluated cognitive function in individuals who had recovered from COVID-19 alongside potentially associated factors such as systemic inflammation [56]. Many COVID-19 survivors showed long-term cognitive impairment in executive function and abnormally elevated plasma TNFα levels were negatively correlated with executive function performance in these individuals. However, in other studies, no significant associations were found between salivary TNFα levels and other factors implicated in poor self-rated health, such as self-rated pain [57].

## 5. Conclusions

The present study is the first to assess the relationship between university students’ sleep quality and their IL-1β levels in saliva. These results are consistent with previous reports conducted with blood samples. Since the collection of saliva samples is simple and non-invasive, this process provides a more convenient method. Moreover, obtaining these samples through chewing the cotton swab instead of stimulating saliva production with citric acid increases the concentrations of most metabolites [58,59]. Although inflammation biomarkers have traditionally been studied in blood samples, IL-1β levels in saliva are highly correlated with serum levels [60]. However, in this work we did not consider oral pathologies, which may influence salivary cytokine levels [61] and could have affected our results by acting as additional confounding factors, however, due to the characteristics of the sample we did not think the number of students with oral pathologies should be relevant over the sample size, but this is to be studied in the future.

Among the psychological components affecting sleep quality, stress level was found to be associated in our study. In this regard, the relationship between the stress hormone, cortisol, and inflammatory markers in the blood demonstrated that HPA axis activity may mediate associations between acute psychosocial stressors and inflammatory processes [62]. However, in more chronic stress conditions, glucocorticoid receptor resistance has been shown to be associated with a failure to down-regulate an inflammatory response [42,63,64]. Given the fact that poor sleep quality was associated with a higher level of stress this could imply that the mechanisms by which inflammatory cytokines increase could be partially mediated by a chronic stress response. This warrants future studies since associations with poor sleep quality in our study were observed for IL-1β but not for IL-6 and TNF-alpha concentrations, suggesting a possible different interplay between cortisol, stress, and each cytokine [65].

Many factors can affect sleep hygiene and thus, sleep quality, but the role of mobile device use in causing sleep problems in adolescence and young adults has gained huge attention in the past few years [66,67]. A recent review reported that one in every four children and young people is suffering from problematic cell phone use, which is linked to depression, anxiety, and poor sleep quality [68,69]. On the other hand, studies such as Rathakrishnan et al. [70] have suggested that smartphone addiction is associated with sleep quality in university students. Therefore, the assessment of these unhealthy habits should be considered in future work. As a limitation of this study, we did not measure the relationship between inflammatory cytokines, sleep quality, and the use of mobile devices at bedtime in the bedroom, which clearly deserves future studies.

Another limitation to this current study was its cross-sectional design because it prevented us from establishing any causal relationships. Moreover, the incidental nature of the sample, which only included nursing students, meant that the cohort was relatively homogeneous and so the study findings cannot be generalized to samples other than nursing students. Thus, in future studies, it would be useful to obtain representative groups of university students through random and stratified sampling and to apply experimental designs to determine causality between the variables studied. It would also be of interest to complement self-reported sleep assessments with objective methods such as actigraphy or polysomnography. However, compared to very expensive objective measures, the questionnaires we used are a useful, simple, and cheap means for assessing variables such as sleep quality.

In summary, our findings suggested that poor sleep quality is positively associated with inflammation, thereby providing a mechanism that could link sleep disturbances to the risk of inflammatory disease. Thus, maintaining a healthy sleep pattern is advantageous for the health of university students who are more likely to have poor quality sleep. Based on this assumption, in addition to contributing to improving knowledge of the underlying pathophysiological mechanisms of poor sleep, the identification of the inflammatory biomarkers of poor sleep quality could be useful to identify people at a greater risk of developing sleep disorders. Likewise, considering that many of the factors related to sleep problems in this population are modifiable through means other than pharmacological therapy, ideally, educational institutions should develop health promotion programs aimed at optimizing sleep habits. Similarly, given that nursing students will go on to work as healthcare providers and caregivers, the promotion of healthy sleep hygiene habits should ideally be included in the curricula of nursing and medical studies.

## Figures and Tables

**Table 1:** Prevalence (reported as a percentage) of the Athens Insomnia Scale (AIS) items (n = 72).

Items of the AIS Scale	No	Slight	Moderate	Severe
Sleep induction	45.8	31.9	22.2	0
Awakenings during the night	44.4	43.1	12.5	0
Final awakening earlier than desired	56.9	36.1	6.9	0
Total sleep duration	31.9	47.2	20.8	0
Overall quality of sleep	47.2	43.1	9.7	0
Sense of well-being during the day	45.8	47.2	5.6	1.4
Functioning (physical and mental) during the day	63.9	31.9	4.2	0
Sleepiness during the day	23.6	59.7	16.7	0

**Table 2:** Prevalence (reported as a percentage) of the Pittsburgh Sleep Quality Index (PSQI) items (*n* = 72).

	Very Good	Fairly Good	Fairly Bad	Very Bad
Sleep quality	29.2	41.7	22.2	6.9
Sleep latency	25.0	33.3	22.2	19.4
Sleep duration	20.8	43.1	31.9	4.2
Habitual sleep efficiency	73.6	9.7	8.3	8.3
Sleep disturbance	6.9	90.3	2.8	0
Use of sleeping medication	76.4	15.3	4.2	4.2
Daytime dysfunction	15.3	43.1	38.9	2.8

**Table 3:** Correlation analyses between sleep quality and psychological assessments.

	Sleep Quality (PSQI)	Self-Perceived Stress (PSS)	Anxiety Symptoms (GADS)	Depression Symptoms (GADS)	Anxiety and Depression Symptoms (Total GADS Score)	Self-Perceived Health
Sleep quality (AIS)						
r	0.723	0.441	0.670	0.478	0.673	−0.355
Sig	<0.001	<0.001	<0.001	<0.001	<0.001	0.003
Sleep quality (PSQI)						
r		0.531	0.498	0.366	0.505	−0.318
Sig		<0.001	<0.001	0.002	<0.001	0.007

Abbreviations: Pittsburgh Sleep Quality Index, PSQI; Athens Insomnia Scale, AIS; Cohen’s Perceived Stress Scale, PSS; Goldberg Anxiety and Depression Scale, GADS.

**Table 4:** Correlation analyses between sleep quality and salivary inflammatory cytokines (interleukin 1 beta, interleukin 6, and tumor necrosis factor alpha).

	Sleep Quality (AIS)	Sleep Quality (PSQI)	Self-Perceived Stress (PSS)	Anxiety Symptoms (GADS)	Depression Symptoms (GADS)	Anxiety and Depression Symptoms (Total GADS Score)	Self-Perceived Health
lL-1β							
r	0.248	0.274	0.054	0.028	0.051	0.044	−0.030
*p*-value	0.038	0.022	0.656	0.818	0.672	0.718	0.805
IL-6							
r	−0.027	−0.055	−0.030	−0.159	0.044	−0.077	0.035
*p*-value	0.822	0.649	0.773	0.188	0.717	0.528	0.773
TNFα							
r	−0.024	−0.035	0.088	0.039	0.117	0.085	−0.259
*p*-value	0.842	0.778	0.470	0.752	0.337	0.490	0.033

Abbreviations: Pittsburgh Sleep Quality Index, PSQI; Athens Insomnia Scale, AIS; interleukin 1 beta, lL-1β; interleukin 6, IL-6; and tumor necrosis factor alpha, TNFα; Cohen’s Perceived Stress Scale, PSS; Goldberg Anxiety and Depression Scale, GADS.

## Data Availability

The data presented in this study are available for scientific purposes on request from the corresponding author.

## Supplementary Materials

The following supporting information can be downloaded at: https://www.mdpi.com/article/10.3390/diseases11040136/s1. Figure S1: Salivary IL-6 (A,B) and TNF-alpha (C,D) concentration in students with normal and poor sleep quality assessed by AIS (A,C) and PSQI (B,D).
