# Supplementary material for: Increased Salivary IL-1 Beta Level Is Associated with Poor Sleep Quality in University Students
Source: Diseases. 2023 Oct 5;11(4):136. doi: 10.3390/diseases11040136 (PMC10594478; doi:10.3390/diseases11040136)

Supplementary Figure S1. Salivary IL-6 (A,B) and TNF-alpha (C,D) concentration in students with normal and poor sleep quality assessed by AIS (A,C) and PSQI (B,D).

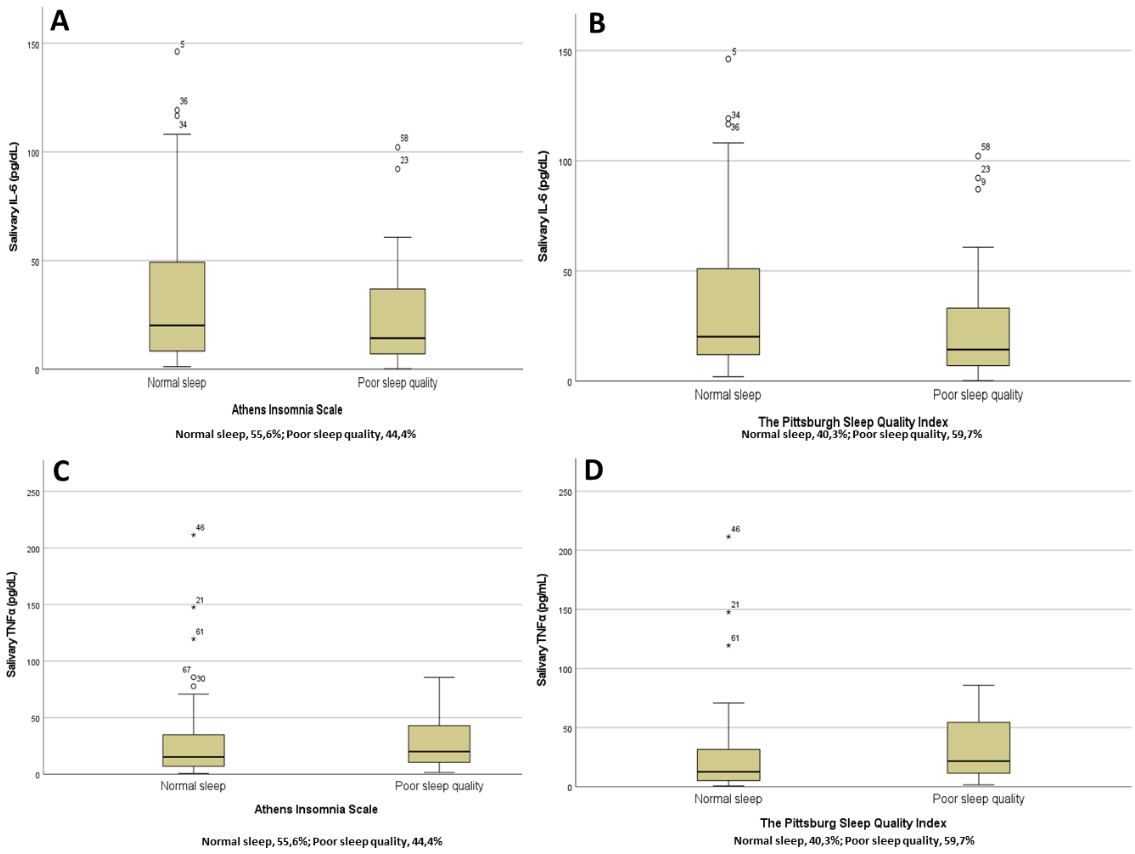

Supplement: Supplementary file 1 [file diseases-11-00136-s001.zip › diseases-2624388-supplementary.pdf]
